# Nigeria′s street children, epitome of oral health disparity and inequality

**DOI:** 10.11604/pamj.2020.36.77.20404

**Published:** 2020-06-09

**Authors:** Enoch Abiodun Idowu, Solomon Olusegun Nwhator, Adedapo Olanrewaju Afolabi

**Affiliations:** 1 Faculty of Dental Sciences, University of Jos, Nigeria,; 2 Faculty of Dentistry, Obafemi Awolowo University, Ile-Ife, Nigeria,; 3 Departement of Dental Services Federal Medical Centre Owo, Ondo State. Nigeria,; 4 Faculty of Dental Sciences, University of Jos, Plateau State, Nigeria

**Keywords:** Street children, Almajiri, oral health awareness, disparity

## Abstract

**Introduction:**

it has been close to four years since the authors highlighted the total neglect of the oral health of street children in the Journal of Public Health Policy. Since then, the authorities appear to have simply turned the blind eye making this follow-up imperative. This follow-up report specifically examines the resultant oral health disparity between Nigeria's street children and Private, fee-paying secondary school students in Northern Nigeria.

**Methods:**

we conducted a cross-sectional comparative survey of randomly selected 12-14 years old street children (children of Quranic informal educational institutions) in Northern Nigeria while fee paying, private secondary school children served as controls. A simple close-ended questionnaire translated into Hausa language was used to assess oral health knowledge and the Simplified Oral Hygiene Index used to categorize oral hygiene status of the participants.

**Results:**

the mean age (SD) of the participating street children was 12.7 (0.86) while that of the private secondary school students (PSSS) was 13.05 (0.96). The majority (89%) of parents of street children compared with that (7%) of parents of students of private secondary schools had no western education. Only 6% of street children compared with 90% of PSSS cleaned their teeth for the right reasons. Only 5% of street children compared with 90% of private secondary school students used a fluoride-containing toothpaste. Though both groups of children have poor knowledge (street children 3%, private secondary school students 16%) on the use of dental floss, the mean oral hygiene score (SD) for street children was 4.42 (0.85) compared with 1.90 (0.09) for private secondary school students. There were striking differences in knowledge and practice with only 4% of street children compared with 69% of PSSS with knowledge about fluoride and its use (p < 0.0005). Also, 2% of street children compared with 40% of PSSS were aware of the benefits of regular dental visits. Sixty five (65%) of street children used finger and water only for tooth cleaning, none of the secondary school students practiced this (p = 0.0005).

**Conclusion:**

there is disparity in oral health practice between Nigeria's street children and private secondary school children. This disparity may be attributed to lack of western education and socio-economic status.

## Introduction

This study is a modest attempt to call attention to the serious oral health disparity between Nigeria's street children and children of comparable age but of a different educational/socio-economic status. Since awareness is fundamental to health-seeking behaviour and utilization, we compared the oral hygiene awareness and practices of 12-14-year-old, street children with those of private secondary school students (PSSS) in the Northwestern city of Kano. Unlike the PSSS, the street children were not exposed to any form of Western education. Education is as old as the human race but means different things to different people being generally a process of teaching and learning for the purpose of acquiring knowledge. With an entry date of 1085-1097 through the Kanem Borno Empire [1], Islamic education predates western education in Nigeria which came in the middle of the 16th century though western Nigeria [2]. Almajiri is an Arabic word derived from Almajihun (searching for Islamic knowledge.) [3, 4]. We already showed that the non-classification of Nigeria's Almajiri as street children constitutes a problem of definition which necessitates a call to action [3]. These Almajiri who should be properly addressed as street children are voluntarily placed in the custody of Islamic teachers called Mallams. Mallams are expected to teach street children the holy Quran but just like their students, the majority of Mallams are unexposed to western education [4]. It is well-documented that parents, siblings, school teachers and socioeconomic factors play important roles in maintaining healthy habits and good oral health [5, 6, 7], the present study investigated the association between these variables. The specific association of interest is the impact of western education of parents on the oral hygiene status, awareness and practices of street children and private secondary school attendees.

Good oral hygiene practice involves maintaining a clean, healthy mouth by brushing and flossing in order to avoid accumulation of debris, plaque and calculus. Ultimately, this is to prevent dental caries and periodontal diseases [8-11].Oral hygiene can in turn, be maintained by good practices like daily brushing with a fluoride toothpaste, flossing with a dental floss, restriction of refined sugar diet as well as regular dental visits and dental education [8-11]. The literature is equivocal on the impact of age and school type on oral hygiene practices [8, 12]. Unfortunately, similar reports on street children are almost nonexistent. What is known is that majority of street children are from socioeconomic backgrounds as poor as those of the Mallams who care for them. These children are left with no option than to embark on unconventional means of survival including street begging [3, 4, 13]. The present study is therefore an attempt to support previous efforts [14-17] to investigate the relationship between socioeconomic background and oral hygiene status. It is worthy of note that the world is now focusing on the oral health of the less-privileged and neglected including street children [3, 4, 13, 18, 19]. Although Nigeria's Almajiri have great resemblance to street children according to United Nation definition [18, 19], debate is still ongoing on categorizing them as street children.

In Northern Nigeria, the general submission is that this group of Nigerian children are from poor homes, uneducated (formal education), vulnerable, neglected and frequently seen roaming the streets of major cities of Northern Nigeria [3, 4, 13, 20]. Street children constitute a large percentage of the child population in Kano city with age range between 5-19 years. This huge population of children has been neglected when planning for comprehensive oral health care for Nigerian children. Generally, there is limited data on the oral hygiene status of school age children in northern Nigeria unlike the southern part of the country where many studies have been conducted among the same population group. Attempts have also been made to compare oral hygiene status and practices of different groups of children in Southern Nigeria [15, 16]. Information on the oral health of school-age children in the Northern city of Kano is scarce and we only recently reported a study on oral health of street children[3]. Presently, there is no study comparing the oral hygiene status of street children with private secondary school students. This study is therefore aimed at comparing the oral hygiene status/awareness and practices of children of different socioeconomic backgrounds in the biggest commercial city in Northern Nigeria.

## Methods

A comparative cross-sectional study of street children and PSSS in Nasarawa Local Government Area (LGA) which was selected by simple random sampling technique among the eight Local Government Areas (LGA) in Kano city. There are many Quranic institutions with a large population of street children as well as private secondary schools in this Local Government Area. The study population comprised of 12-14year old street children and private secondary school students. The calculated sample of 400 (200 per group) was based on the formula proposed by Machin and Campbell in 2011 [21]. While all participating street children in this study were male, both male and female PSSS participated. Multistage random sampling technique was adopted for this study using the list of registered private secondary schools available at the Local Government Educational Authority. There was no list of Quranic institutions with the Local Authority hence the need to generate one from two randomly selected wards out of the thirteen in the LGA. From this generated list, 10 Quranic centers were randomly selected with 200 street children. Out of the sampling frame of the list of nineteen registered private secondary schools in the LGA, three were randomly selected. In each of these schools, the junior secondary classes with pupils within the age bracket of the study population were identified and based on the populations of the pupils in each school, (90, 70, and 40) students were randomly selected to participate in the study.

Before the commencement of the study, clearance was obtained from the ethics committee of Aminu Kano Teaching Hospital, Educational Department of Nasarawa LGA and Management Authority of each participating school. The parents, *Mallams* and/or guardians of the private secondary school students and street children gave written informed consent while the students and street children gave verbal assent. All information was translated/explained in Hausa language for those who had language challenges. To be included, children had to be 12-14 years old, willing to participate in the study and to undergo simple clinical oral examination. Consequently, children below 12 or above 14 years of age, parents not willing to sign informed consent and those students and street children who declined assent to undergo simple clinical oral examination were excluded from this study. Data for this study were collected using close-ended, examiner-administered questionnaires and simple clinical oral examinations. The semi-structured questionnaires were available in English and Hausa languages and were administered by the leading researcher assisted by two dental therapists. The questionnaires were administered to pupils who filled the questionnaires at the same time (simultaneously) to avoid bias. The questionnaires were divided into three parts including a bio-data and parental educational background section, an oral health awareness assessment section and an oral hygiene practice section. The questionnaire was pretested in a pilot study conducted in two schools with similar socio- demographic characters as the target study participants. The responses obtained from the participants in this study were similar to those of the pilot study. The simplified oral hygiene index of Green and Vermillion was adopted for assessing oral hygiene status of the subjects in this study using six index teeth namely 11, 16, 26, 31, 36 and 46. Oral hygiene index scores of 0-1.2 were classified as good, 1.3-3.0 as fair and 3.1 - 6 as poor.

Two calibrated examiners performed clinical oral examinations under natural light while two trained dental therapists recorded findings. The materials used for clinical oral examinations included dental mouth mirrors, wooden spatulas, caries probes, college tweezers, cotton wool, dispensable plastic cups, face masks, disclosing tablets, hand towel, water and autoclaving machine. A pilot study was conducted and inter rater reliability performed prior to the main study with good reliability (k=0.8). The data collected were coded while analyses performed using SPSS version 15.0 to generate frequencies/proportions and means. Student t- test was used to compare means while chi square test was used to compare proportions at 95% confidence level. Therefore, p values of ≤0.05 were considered significant.

## Results

Four hundred children participated in this study consisting of an equal number (200) of street children and private secondary school students. All the street children that participated in this study were males while the PSSS were both males and females. In this present study, sexes of the participants were not considered because previous studies have indicated similarities in oral health awareness among boys and girls [21, 22]. The male to female ratio was 3:1. The mean age (SD) of the participants was 12.9 (0.93) years with the street children being 12.7 (0.86) and PSSS being 13.05 (0.96) (Table 1). While a greater percentage of parents of PSSS were exposed to western education at different levels, most parents of the street children´ had never been exposed to ‘formal´ western education. It was observed that 28% (n=56) and 89% (n = 178) of PSSS mothers and fathers respectively, have been educated to the tertiary level of western education. On the other hand, none, 0% (n = 0) and 1% (n = 2) street children mothers and fathers respectively, had been exposed to any form of western education. Socio-demographic characteristics of the street children in this study were inconclusive because more than 80% of them could not identify their parents' occupation while 75% of PSSS stated that their parents were civil servants.

**Table 1:** shows the demographic details and educational levels of parents of participants

Variables	Street Children		Private		Total	
	n=200		n=200		n=400	
**AGE Mean (SD)**	12.70 (0.86)		13.05 (0.96)		12.90(0.9)	
	**Freq**.	**%**	**Freq**.	**%**	**Freq**.	**%**
**GENDER**						
Male	200	50	100	60	320	80
Female	0.0	0.0	80	40	80	20
**Total**	200	100	200	100	400	100
**Mother's Education**						
None	180	90.0	8	4	188	47
Primary	12	6.0	8	4	20	5
Secondary	8	4.0	128	64	136	34
Tertiary	0.0	0.0	56	28	56	14
**Total**	200	100%	200	100%	400	100%
**Father's Education**						
None	176	88.0	6	3	182	45.5
Primary	10	5.0	8	4	18	4.5
Secondary	12	6.0	8	4	20	5
Tertiary	2	1.0	178	89	180	45
**Total**	200	100%	200	100%	400	100%

One hundred and forty students (70%) of PSSS agreed with the two important reasons behind regular teeth cleaning (i.e. prevention of infection and mouth odor), only 6% (n = 6) of street children agreed with this. Also, majority of street children representing 57% (n = 114) in contrast to 16% (n = 32) of PSSS stated mouth odor as the only reason for cleaning their teeth regularly. On the awareness of fluoride and its use, 4% (n = 8) of street children compared with 69% (n = 138) of PSSS were aware of the oral health benefits of fluoride application and usage. The findings were statistically significant (p=0.0005). Many participants in this study were aware of the harmful effect of frequent and uncontrolled intake of refined sugar on their teeth, representing 65% (n = 130) of street children and 91% (n=182) of PSSS. Table 2 showed that in both groups majority of participants approximately 79% (n = 316) were bordered about the negative impact of tooth decay. However, this awareness was observed more among the street children 84% (n = 168) compared with PSSS 74% (n = 148). Both groups of children were also well-aware of important roles of teeth in the body. The response to questions on the need for regular dental visit among the pupils showed that only 2% (n = 5) of street children and 40% (n = 80) of private school children were well-aware of the benefits of regular dental visits.

**Table 2:** shows the responses of participants to oral health knowledge and awareness questions

Variables	Street Children		Private		Total		P. Value
	**Freq**.	**%**	**Freq**.	**%**	**Freq**.	**%**	
**TOOTH CLEANING REASON**							
I don't Know	24	12	2	1	26	6.5	
To prevent odor	114	57	32	16	146	36.5	0.00(S)
To prevent infection & odor	12	6	140	90	152	38.0	
**Total**	**200**	**100%**	**200**	**100%**	**400**	**100%**	
**FLOURIDE USE**							
Yes	192	96	62	31	254	63.5	0.00(S)
No	8	4	138	69	146	36.5	
**Total**	**200**	**100%**	**200**	**100%**	**400**	**100%**	
**SUGAR & TOOTH DECAY**							
No	70	35	18	9	88	22	
Yes	130	65	182	91	312	78	
**Total**	**200**	**100%**	**200**	**100**	**400**	**100%**	
**NEGATIVE IMPACT OF TOOTH DECAY**							
I don't know	16	8	40	20	56	14	
Bothered	168	84	148	74	316	79	
Not Bothered	16	8	12	6	28	7	0.00(S)
**Total**	**200**	**100%**	**200**	**100%**	**400**	**100%**	
**TEETH-BODY PARTS**							
No	10	5	4	2	14	3.5	
Yes	185	92.5	192	96.0	377	94.2	
I don't know	5	2.5	4	2	9	2.2	
**Total**	**200**	**100%**	**200**	**100%**	**400**	**100%**	
**PERIODIC DENTAL VISITS**							
Yes	195	97.5	120	60	315	78.8	
No	5	2.5	80	40	85	21.2	
**Total**	**200**	**100%**	**200**	**100%**	**400**	**100%**	

In response to the question on frequency of tooth cleaning, 35% (n = 70) of street children compared with 0% (n = 0) of PSSS cleaned their teeth 5 times a day. Whereas 49% (n=98) of street children and 45.5% (n=91) of PSSS cleaned their teeth twice daily, 13% (n = 26) of street children and 54% (n = 108) of PSSS cleaned their teeth only once a day. Only 3% (n = 6) of street children with 0.5% (n = 1) of PSSS rarely cleaned their teeth. Regarding tooth cleaning time, 16% (n = 32) of street children compared with 59% (n = 118) of PSSS clean their teeth before breakfast while 37% (n=74) of street children and 10% (n=20) of PSSS cleaned their teeth after each meal. A total of 34% (n = 68) and 0% (n = 0) of street children and PSSS respectively, cleaned their teeth only at prayer time. Table 3 also shows that only 3% (n = 6) of street children compared with 16% (n = 32) of PSSS have ever used dental floss for inter dental cleaning.

**Table 3:** shows different methods of oral hygiene practice among the study groups

Variables	Street Children		Private		Total	
	**Freq**.	**%**	**Freq**.	**%**	**Freq**.	**%**
**Cleaning Frequency**						
Rarely	6	3	1	0.5	7	1.8
Once a day	26	13	108	54	134	33.5
Twice a day	98	49	91	45.5	189	47.2
Five times a day	70	35	0.0	0	70	17.5
**Total**	200	100%	200	100%	400	100%
**TIME OF CLEANING**						
Before breakfast	32	16	118	58	150	37.5
After every meal	74	37	20	10	94	23.5
Prayer time alone	68	34	0	0	68	17.0
Before breakfast and night	22	11	60	30	82	20.5
At night alone	4	2	2	1	6	1.5
**Total**	200	100%	200	100%	400	100%
**CLEANING MATERIALS**						
Fluoride containing tooth	10	5	180	90	190	47.5
Tooth brush alone	26	13	14	7	40	10
Chewing stick	34	17	5	2.5	39	9.8
Water and finger	130	65	0	0	130	32.5
Chlorhexidine solution	0	0	1	0.5	1	0.2
**Total**	200	100%	200	100%	400	100%
**DENTAL FLOSS KNOWLEDGE**						
No	194	97	168	84	362	90.5
Yes	6	3	32	16	38	9.5
**Total**	200	100%	200	100%	400	100%

A greater percentage of street children have never come across or used dental floss as an aid for tooth cleaning, compared with PSSS (p = 0.0005). Likewise, 5% (n = 10) of street children compared with 90% (n=180) of PSSS used fluoride- containing toothpaste. Again, 13% (n = 26) of street children compared with 7% (n = 14) of PSSS used tooth brush alone for cleaning their teeth and 65% (n = 130) of the street children used their fingers and water only for cleaning their teeth compared with 0% (n = 0) of PSSS. This finding was statistically significant (p=0.0005). Table 4 shows that only 2.0% (n = 4) of street children compared with 64.0% (n = 128) PSSS had good oral hygiene, while 49.0% (n = 98) of street children had poor oral hygiene compared with 3% (n=6) PSSS (p = 0.000). The mean (SD) oral hygiene index score for the street children was 4.42 (0.85) while that of PSSS was 1.9 (0.09). Considering the overall result of the oral hygiene index score as observed in this study, majority of the participants had fair oral hygiene representing 41.4% with mean (SD) oral hygiene score of 3.14 (0.91).

**Table 4:** shows the oral hygiene status of the study population

Scores	Oral hygiene	Street Children		Private		Total	
		**Freq**.	**%**	**Freq**.	**%**	**Freq**.	**%**
**0-1.2**	Good	4	2	128	64	132	33
**1.3-3.0**	Fair	98	49	66	33	164	41
**3.1-6**	Poor	98	49	6	3	104	26
**Total**		200	100%	200	100%	400	100%

## Discussion

All the street children that participated in this study were males unlike the PSSS that were males and females. The sex of the participants was, however, considered insignificant because similar studies have shown that sex plays no role in oral health awareness and oral hygiene practices among subjects of same age group [22, 23]. The second reason was that the street children system originally excludes the female sex. Currently, the elite are confused regarding reports about the rise in female street children. This confusion however arises from a misunderstanding of the street children system. The definition of the USAID [24], however, solves the puzzle, defining street children as “a Hausa term that refers to children who have moved away from home to live with a *mallam* (or Quranic teacher) for full-time study of the Quran.” The sighting of “female” street children in Quranic schools has been confused with the introduction of female street children, but by definition, attendance at a Quranic school does not automatically translate to being a street child unless such girls have actually “moved away from home to live with a *mallam* (or Quranic teacher) for full-time study of the Quran”.

We observed that both PSSS and their parents were exposed to western education at various levels but apart from the fact that all the street children were not exposed to western education. The significant higher level of oral health awareness among PSSS compared with street children in this study is consistent with previous reports [25, 26]. This result is also consistent with previous reports that parents, siblings and school teachers play an important role in growing healthy habits in young children [7, 27]. The case of the street children may have been compounded by the lack of exposure to western education by their *mallams* (guardians) and parents .The poor oral health awareness mostly noticed among street children is consistent with previous studies among children of low socioeconomic background[25, 26]. The findings are also consistent with the reports of previous studies among school children of similar age group in developing countries with large populations like India and China [22, 23, 28]. This is attributable to factors like ignorance, poor oral hygiene practices and poverty as a result of poor family income.

Participants' response to major reasons for cleaning their teeth in this study showed that majority (57%) of the street children clean their teeth only to prevent mouth odor compared with a greater percentage (70%) of the PSSS who cleaned their teeth in order to prevent mouth odor and dental diseases. This finding corroborates a previous report[29]. Again, the PSSS were more aware that fluoride use can prevent tooth decay. In the present study, regular use of fluoride among street children was much lower than among PSSS. Despite their better use of fluoride toothpaste, however, the level of fluoride toothpaste use among the PSSS was lower than reported in a previous Indian study [22] but compares favorably with fluoride toothpaste use of 94.8% reported among children in Enugu-Nigeria [23].

In this study, both groups were well-aware of the harmful effects of frequent and indiscriminate consumption of refined sugar on their teeth. There was no significant difference in their response (p>0.05). It was equally observed that majority of both groups were aware of the negative impact of tooth decay on their health and that teeth were as important as any other part of the body. The awareness of the need for dental visits was higher among PSSS, corroborating previous studies [26, 30] and is consistent with previous studies among children of similar age group [8, 12, 31-34]. Furthermore, we found that 34% compared with 67% from a previous study cleaned their teeth at least once daily [8]. It is noteworthy that some street children (35%) claimed to clean their teeth five times daily, compared with 0.0% of PSSS. This act of five times daily cleaning by street children was part of a religious practice rather than deliberate oral hygiene practice. The clearer picture on deliberate oral hygiene practice of tooth brushing was higher among PSSS. The finding is consistent with a previous report [12] but at variance with another study which observed no significant relationship between type of schooling, age and habit on tooth brushing [8]. The percentage of the study population that cleaned their teeth twice a day and after meal in the morning and night which is the ideal method was found to be lower in this present study than in the previous studies among a similar age group in Europe [32, 35], the Middle East [33-36] and China [30, 32, 35, 37].

Majority of the street children (65%) used fingers and water only for cleaning their teeth, a practice found to be nonexistent among PSSS. While only 5% of the street children used toothpaste and toothbrush for tooth cleaning, a higher percentage (90%) of PSSS used toothpaste and tooth brush for regular tooth cleaning due to exposure to proper form of tooth-cleaning. The proportion of the PSSS using toothpaste and toothbrush for tooth-cleaning was higher than what was reported in previous studies among a similar age group of children in China [25]. Other tooth-cleaning materials used by the respondents were chewing stick and toothbrush without toothpaste. The percentage of children in this study that had ever used dental floss for inter-dental cleaning were very low and consistent with previous studies [25, 38]. Studies have shown that effective oral cleanliness can only be achieved with the use of toothbrush and fluoride-containing toothpaste and not with water and finger as was common among the street children [39-41]. This strange form of oral hygiene practice among street children may be associated with poverty, ignorance and lack of western education. This might also have contributed significantly to the higher proportion of street children that presented with poor oral hygiene compared with PSSS in this study.

In summary therefore, this study unearthed important paradoxes among the study population and gross oral health inequalities. First, regarding tooth-cleaning and oral hygiene, while 35% of street children claimed to clean their teeth five times daily, compared with 0.0% of PSSS but the same tooth-cleaning street children had significantly poorer oral hygiene than the PSSS (p=0.000). With only 2.0% (n=4) of street children compared with 64.0% (n = 128) of PSSS having good oral hygiene, frequent tooth cleaning did not seem to translate to better oral hygiene among the street children. The paradox, however, gets unraveled with the discovery that only 5% of the street children actually used tooth brush and tooth paste for tooth cleaning compared with 90% of PSSS. Most (65%) (n = 130) of the street children actually used finger and water only for cleaning their teeth-a practice absent among PSSS which clearly solves the paradox. Secondly, the street children were seemingly more concerned about tooth decay than the PSSS but they (street children) almost never used fluoride toothpaste because of low awareness and poverty. It was again paradoxical that majority of both groups were aware of the negative impact of tooth decay on their health and that teeth are as important as any other part of the body. Surprisingly, this level of awareness did not translate to regular dental visits among the subjects. Finally, even with the awareness of the benefits of regular dental visits among 80% of PSSS did not translate into actual dental visits among them highlighting the limitations of self-reported claims of awareness.

**Recommendations:** periodic oral health awareness campaigns targeted at street children should be organized by community health extension workers or where available, the primary oral health care providers by the Local Government Authorities and the Kano State government. Both the *mallams* and the secondary school teachers should be specially educated on oral hygiene practices. Since majority of the *mallams* act as both foster parents and teachers to the street children, giving them a certain degree of training in oral health awareness may in turn help their students to be better informed. Oral health care delivery should be made more accessible to the Nigerian children through the establishment of mobile dental clinics that will pay periodic visits to Quranic and formal schools.

## Conclusion

The level of oral disparity and resultant inequality suffered by Nigeria's street children is unacceptable. These street children are regarded as the scum of the society, neglected and abused. Nobody wants to be associated with their problems or read about them in journals. We found that unlike the street children, PSSS with good western educational background were aware of their oral health which in turn translated to better oral hygiene practices and better oral hygiene status. Therefore, though the influence of socioeconomic factors on oral health remains inconclusive in the literature, educational exposure may well play a significant role in oral health awareness/practice and oral hygiene status. Policy makers cannot wish the problem away or sweep it under the carpet while turning the blind eye, the academia and academic publishers must give priority to this silent inequality.

### What is known about this topic

- Almajiris share lots of characteristics with street children;
- Most Nigeria street children live in Northern Nigeria under deplorable conditions and deprivations;
- Street children suffer tremendous neglect of their general health.

### What this study adds

- Street children suffer poor oral health;
- Oral hygiene practices among street children is still rudimentary, primitive and ineffective;
- Oral hygiene habits of street children is worse than those of children of comparable age in private secondary schools.
